# Pregnancy through the Looking-Glass: correlates of disordered eating attitudes among a sample of Lebanese pregnant women

**DOI:** 10.1186/s12888-023-05205-w

**Published:** 2023-09-26

**Authors:** Sarah Gerges, Sahar Obeid, Souheil Hallit

**Affiliations:** 1 School of Medicine and Medical Sciences, Holy Spirit University of Kaslik, P.O. Box 446, Jounieh, Lebanon; 2 Social and Education Sciences Department, School of Arts and Sciences, Lebanese American University, Jbeil, Lebanon; 3 Research Department, Psychiatric Hospital of the Cross, Jal Eddib, Lebanon; 4 Applied Science Research Center, Applied Science Private University, Amman, Jordan

**Keywords:** Eating disorders, Pregorexia, Mental health, Mental disorders, Body image, Pregnancy

## Abstract

**Introduction:**

Despite the risks of gestational disordered eating for both the mother and fetus, research into this subject is scarce within developing countries, particularly in Lebanon. Our study’s objective was to delve into the predictors of disordered eating attitudes during pregnancy among a sample of Lebanese pregnant women while assessing the potential mediating effect of body dissatisfaction between psychosocial factors and disordered eating attitudes in pregnancy.

**Methods:**

We framed a cross-sectional study, built on self-report measures. Pregnant women of 18 years old and above were recruited from all the Lebanese governorates through an online survey (*N* = 433).

**Results:**

The results showed that higher pregnancy-specific hassles (Beta = 0.19), media and pregnant celebrities’ influence (Beta = 0.22), and body dissatisfaction (Beta = 0.17) were significantly associated with increased disordered eating attitudes in pregnancy; whereas higher perceived social support (Beta = -0.03), lower socio-economic status (Beta = -0.84), and multigravidity (Beta = -0.96) were significantly associated with less disordered eating attitudes during pregnancy. Body dissatisfaction mediated the association between pregnancy-specific hassles and disordered eating attitudes, and between social appearance concerns and disordered eating attitudes.

**Conclusion:**

Our study highlighted that antenatal care, particularly in Lebanon, should no longer be limited to biological monitoring but rather seek to identify possible eating disorders and mental health threats. Further investigations following longitudinal designs should pursue identifying additional correlates of gestational disordered eating in the clinical context, in furtherance of consolidating screening programs and building targeted treatment strategies.

**Supplementary Information:**

The online version contains supplementary material available at 10.1186/s12888-023-05205-w.

## Introduction

The exploration of eating disorders during pregnancy, such as anorexia nervosa, bulimia nervosa, binge eating disorders, and Other Specified Feeding and Eating Disorders (OSFED), has always been a subject of great interest in both research and clinical fields [1–4]. However, a peculiar designation of disordered eating attitudes during pregnancy has only begun to rise in attention few years ago—which is “Pregorexia”. In 2008, this concept emerged for the first time through the popular media, (1) attributing the term pregorexia to an irrational anguish of the natural weight gain in the course of pregnancy and an exhausting desire to remain svelte—cost what it may—and (2) describing dangerous measures pregnant women would adopt to strictly restrict extra-kilograms, namely extremely dieting, starving, and spending several hours a day strenuously exercising [5].

To date, pregorexia has neither been officially adopted as a medical term nor classified in the Diagnostic and Statistical Manual of Mental Disorders, Fifth Edition – Text Revision (DSM-5-TR) eating disorders criteria [6]. Although this condition is not yet recognized diagnostically, it shares facets with other types of eating disorders, specifically anorexia nervosa. Given that women with pregorexia may experience eating restriction as well as bingeing followed by purging [7, 8], we might simply define it as anorexia nervosa that manifests for the first time during pregnancy [5]. In fact, the neologism “pregorexia” results from the combination of two words (“pregnancy” and “anorexia”), in order to designate anorexia-like symptoms encountered during pregnancy—which are specifically related to the changes endured by the pregnant body [7].

One of the factors that may significantly contribute to this condition is the fact that young women are nowadays perpetually coerced into maintaining sylphlike silhouettes by a variety of societal components (i.e., family members, friends, social media, and social culture of thinness) [9]. Deeply in our human consciousness, weight, body size, and body shape (e.g., waist-to-hip ratio) constitute the primary determinants of women’s physical attractiveness [9]. A meta-analysis of seventy-seven studies found that exposure to media images dictating subconscious internalizations of the ideally-thin female body predicted greater overall levels of vulnerability to body shape concerns among women [10]. A recent review has also emphasized the specific role of self-comparison to slender celebrities in the establishment of negative portrayals of one’s physical appearance [11]. Moreover, in an era where even pregnancy remains mediatized, the distortions of reality by overly thin pregnant celebrities and the constant promotion of “glamour pregnancies” have extended the excruciating preoccupations about body image to the pregnancy and postpartum periods [12]. In addition to the pressures exerted by the society and popular media platforms, it has long been postulated that women who are predisposed to pregorexia lack adequate social support systems [5].

Besides, the phenomenon of pregnancy by its very nature—especially primigravidity—has been numerously described as a “psychological burden” [13]: each pregnancy carries intense cognitive fluctuations and emotional disturbances, then turning into an intrinsically potent stressor [13] that might trigger new-onset types of eating disorders. Regardless of the previous status of eating disorders and the appropriateness of gestational weight gain, women have stressed that pregnancy definitely impacts self-image and emotions, hence inducing focus on food as a coping strategy for anxious states of mind [5].

Conversely, many investigators have witnessed attenuations of disordered eating symptoms during pregnancy compared to pre-conception [14–18]. In point of fact, a considerable proportion of women regarded pregnancy as a propitious occasion to get relief from social norms of slimness [19], even though a study concluded that this effect appeared to be state-dependent and was far from reflecting a change in body standards or a definite deliverance from sociocultural norms of thinness [20].

Be that as it may, there is evidence that eating disorders may persist into pregnancy [21]. Additionally, when pursuing the evolution pattern of body image perceptions prior to pregnancy through the post-partum period, Coker and Abraham discovered that body weight dissatisfaction failed to improve during pregnancy for both the eating disorder and control groups, remaining unchanged for the former and up-surging in the latter. On top of that, it persisted high for the control group until 6 months postpartum [22]. As a result, their research validated the speculation that confers to pregnancy the power to instigate weight concerns and disorders.

In light of these facts, it has become entrancing to elucidate the psychopathological models underlying gestational disordered eating. For this purpose, researchers have been sparsely investigating this condition over the past few years; highlighting the interrelation between mental health disorders, namely depression and anxiety, psychosocial factors such as maternal attitudes towards pregnancy/motherhood and social support, and disordered eating throughout pregnancy [23–25]. Consequently, they have accentuated the importance of inquiring into the associations between psychosocial factors and gestational disordered eating. However, factors increasing the tendencies of partaking in perilous eating patterns during pregnancy have not been thoroughly investigated.

In fact, the literature has long tackled the associations of disordered eating attitudes with appearance-related concerns, body dissatisfaction, and thin-ideal representations caused by the media. In this regard, researchers were successful in establishing solid theoretical frameworks among female adolescents and young adults. For instance, a study demonstrated that appearance-focused social media use and greater “photo activities” were related to heightened body image concerns, body surveillance, and thin-ideal internalizations in young women [26]. Likewise, another investigation discovered that body image, social media, and the desire to achieve an overly thin body all impacted disordered eating attitudes among female university students [27]. Similarly, prior research suggested that media-related thin internalizations educe body dissatisfaction, which—in turn—drives to disordered eating attitudes, particularly restrained eating and weight disorders [28]. Nevertheless, the validity and applicability of these theories during pregnancy remain equivocal, especially when taking into account the predictive influences of social appearance concerns and media/pregnant celebrities on disordered eating attitudes, as well as the mediating effect of body dissatisfaction within these relationships.

In line with this perspective, a qualitative analysis of pregnant women's body image experiences revealed that they were perplexed; precisely, their body perceptions were contingent on their expectations regarding the future prominence of their body parts during the perinatal period, as well as their attitudes towards maternity clothing. However, the recognition of the pregnant body’s functionality was able to enhance positive feelings instead [29]. Consequently, it is conceivable that the mother’s appraisal of the various emotional stimuli during pregnancy may influence her satisfaction with her body. It is therefore plausible that women who feel more hassled by pregnancy are more likely to develop body dissatisfaction, hence engaging in disordered eating attitudes and weight-restrictive behaviors. Nonetheless, to our best knowledge, no previously conducted research has considered the mediating role of body dissatisfaction in the association of pregnancy-related stress with disordered eating attitudes in pregnancy.

Since the relative contributions of psychosocial factors in the prediction of eating disorders during pregnancy remain insufficiently understood, exploring the correlates of disordered eating attitudes in pregnancy and studying mediating factors may deliver insightful information about the psychopathology of these conditions and thus refine screening and treatment procedures. Such contributions are of utmost importance, as researchers have identified a wide number of severe complications induced by gestational undernutrition over the years; including maternal anemia/vitamin deficiencies, fetal growth restriction/impaired development, as well as short stature, obesity, strokes, hypertension, coronary heart disease, metabolic syndrome, and non-insulin dependent diabetes in adulthood [30–32]. Likewise, eating disorders during gestation do not refrain from afflicting women's mental health, and several studies underlined remarkable interactions between depressive and anxiety symptoms during pregnancy and the disordered eating symptomatology [4, 33, 34]. Furthermore, it is estimated that 5% of women experience pregorexia during pregnancy and after delivery [35]. These evidence assign to gestational anorexia-like disordered eating (i.e., pregorexia) a global and public health dimension, and account for its noteworthiness as a perilous legitimate disease.

**Fig. 1:**
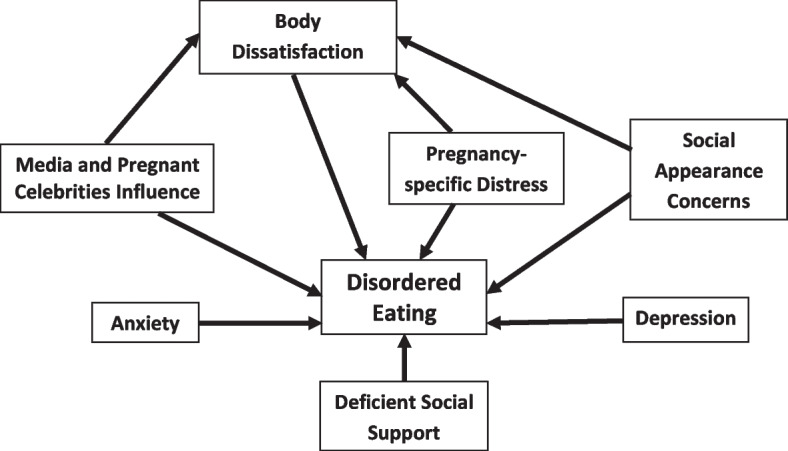
Conceptual model of factors associated with disordered eating attitudes during pregnancy

Even though obesity during pregnancy is considerably more common than pregorexia [5], food and nutrition experts, gynecologists, psychologists, and psychiatrists should be alert for any indications that a pregnant woman is unduly preoccupied with her body image rather than her adequate nutrition and health during pregnancy. Given that this subject has received the least attention in developing and Middle-Eastern countries [36], particularly during pregnancy, it becomes all the more important to conduct such a study in Lebanon, a Middle-Eastern developing country. Therefore, the current study aimed to examine the correlates of disordered eating attitudes among Lebanese pregnant women, and was thus built on a conceptual model of psychological and psychosocial factors that may be linked to disordered eating during pregnancy (Fig. 1). On account of the aforementioned evidence, we hypothesized that being more socially supported would be associated with less disordered eating attitudes in pregnancy, whereas all the remaining illustrated factors would increase the propensities for such attitudes. We also anticipated that body dissatisfaction would play a key mediating function in the associations between psychosocial factors and disordered eating attitudes during pregnancy.

## Methods

### Study design

We framed a cross-sectional study, built on self-report measures. Pregnant women of 18 years old and above were recruited from all the Lebanese governorates (i.e., Beirut, Mount Lebanon, North, South and Bekaa) during June and July 2021. They were asked to fill out an online questionnaire, shared on social media networks (e.g., WhatsApp and Facebook), and spread it among other pregnant women as well. Anonymity was guaranteed. The online snowball technique (via social media platforms) that we followed for the data collection process was in conformity to the COVID-19 pandemic-induced governmental restrictions, which were the imposed lockdown and the impossibility of conducting face-to-face interviews, and whose aim was to reduce the risks for both interrogators and participants.

### Minimal sample size calculation

When accepting a type I error (α) of 5% and a power (1-β) of 80%, the G*power 3.1.9.7 software (linear multiple regression: fixed model, R^2^ increase) [37] indicated that a minimal sample size of 395 was compulsory to achieve significance for an effect size (F^2^) of 0.02 (small effect, as categorized by Cohen [38]) and a total number of predictors *N* = 10 to be considered in the final multivariable model. Ultimately, 433 pregnant women took part in our study.

### Translation procedure

Apart from the Lebanese Anxiety Scale (LAS-10) [39] that was constructed and validated in Lebanon, and the Arabic version of the Patient Health Questionnaire (PHQ-9) that has previously shown its reliability among the Lebanese population [40], all scales underwent two consequential translations. The procedure was initiated by a forward translation, from English to Arabic, performed by a bilingual healthcare professional. Then, it was completed by a backward translation into English, performed by another bilingual primary care provider who was blinded to the scales’ notions and their initial English versions. This process strictly follows international guidelines for a pertinent cultural adaptation of self-assessment scales by and large, and clinical health measures in particular [41, 42]. No discrepancies were noted in terms of intellectual consistency. At the end, two psychiatrists and two psychologists revised and agreed to the measures’ final versions [43, 44].

### Questionnaire and variables

The questionnaire was following a self-administration mode of closed-ended questions written in Arabic, the mother tongue of Lebanon. It was divided into two major parts requiring approximately fifteen minutes. Participants were instructed to fill it out without assistance, in order to avoid any social pressure or undesirability. The first part included questions assessing socio-demographic variables: age, marital status, residency area (governorate), religion, educational level (categorized into complementary, secondary and university level), as well as the socioeconomic status (estimated by the household crowding index). This unit of measurement relates the number of the participant’s family members living under the same roof (including herself) to the number of rooms forming her household (excluding the kitchen and bathrooms). The higher the obtained ratio, the lower her economic status [45]. Pregnant women were also asked to report their gestational age (i.e., pregnancy week) at the moment of participation. Gravidity (i.e., number of the current pregnancy) was categorized into “first pregnancy” (primigravida), “second pregnancy” (secundigravida) and “third pregnancy or more” (multigravida). Height and weight (i.e., pre-pregnancy weight and current weight) were collected. The Body Mass Index (BMI) was calculated by dividing the weight in kilograms by the height in meters squared. The physical activity index was evaluated considering the intensity, duration and frequency of exercising during pregnancy [46].

The second part comprised several scales for a relevant assessment of our variables of interest:

#### The Arabic version of the Disordered Eating Attitudes in Pregnancy Scale (A-DEAPS)

The A-DEAPS is a rapid and useful screening tool based on a Yes or No type of questions. This instrument has been validated in Lebanon [47]. It comprises ten statements illustrating disordered eating attitudes in pregnancy, including cognitive distortions (e.g., “I have felt anxious about eating in general, or about eating certain foods” and “I have wanted my pregnancy body to be small, like I am “just bump” (i.e., only my stomach appears to have grown, with no weight or shape changes to other areas of my body)”), as well as toxic behaviors such as “I have attempted to stop the changes occurring to my body during pregnancy”, “I have noticed that what I allow myself to eat and how much I can eat is connected to rules and conditions” and “I have spent considerable time researching the most effective ways to minimize how much weight I gain while pregnant". The original DEAPS was developed by Bannatyne et al., who aimed to provide a reliable instrument to screen for pregnancy-specific symptoms of disordered eating [48]. The A-DEAPS scores range between 0 and 10. A higher score reflect more disordered eating attitudes during pregnancy (worries about weight gain in pregnancy, food anxiety, and calorie restriction). In this study, the Cronbach’s alpha = 0.805 in the total sample.

#### The Patient Health Questionnaire (PHQ-9)

In its original validation, this brief tool was of a great efficacy in detecting depressive disorders among clinical samples, and particularly in patients recruited from the gynecology/obstetrics context. Each one of its nine items (e.g., “Little interest or pleasure in doing things” and “Feeling down, depressed, or hopeless”) is scored as 0 (not at all) to 3 (nearly every day), thus quantifying the severity of symptoms [49]. This scale has also been validated in Lebanon [40]. In our study, the Cronbach’s alpha = 0.846.

#### The Lebanese Anxiety Scale (LAS-10)

The LAS-10 is a brief and efficacious scale, whose ten items arose from the DSM-5, HAM-A, and STAI measures’ pool of diagnostic criteria. To exemplify, items include “I have an anxious mood (worries, anticipation of the worst, fearful anticipation, irritability)” and “I feel that difficulties are piling up so that I cannot overcome them”. It was purposely developed and validated to screen for anxiety among the Lebanese population [39]. The higher the score, the more intense the anxiety. In this study, the Cronbach’s alpha = 0.897.

#### The Pregnancy Experience Scale – Brief form (PES-Brief)

The PES-Brief is the short form of the original PES scale that contains two subsets of items. The PES-Uplifts appraises the intensity of maternal joyfulness towards the positive experiences that are exclusively encountered in pregnancy; it comprises ten items, including “How much the baby is moving”, “Making or thinking about nursery arrangements”, and “Visits to obstetrician/midwife”. On another hand, the PES-Hassles quantifies the pregnant woman’s annoyance engendered by a variety of pregnancy-specific hassles through ten additional items that include “Normal discomforts of pregnancy (heartburn, incontinence)”, “Thinking about your labor and delivery” and “Thoughts about whether the baby is normal”. Scores fall between 0 “not at all” and 3 “a great deal” for each item, and total scores range from 0 to 30 for both PES-Uplifts and PES-Hassles. Higher PES-Hassles scores and lower PES-Uplifts ones predict greater maternal distress in response to negative and positive pregnancy-related emotional stimuli, respectively [50]. In this study, Cronbach’s alpha = 0.868 (PES-Uplifts) and Cronbach’s alpha = 0.809 (PES-Hassles).

#### The Body Dissatisfaction scale of the Eating Disorder Inventory (BD-EDI)

This 9-item subscale is a component of the large EDI-2 that assesses the psychology of eating. The body dissatisfaction scale’s items (e.g., “I think my buttocks are too large”) are scored from 0 (sometimes/rarely/never) to 3 (always), with five reversed questions (e.g., “I feel satisfied with the shape of my body”) and a possible total score falling between 0 and 27. The higher the score, the lesser the pregnant woman’s satisfaction with her body [51]. Cronbach’s alpha in this study = 0.808.

#### The Multidimensional Scale of Perceived Social Support (MSPSS)

It is a succinct research instrument, gauging the degree of individual perceptions of social support that emanates from three distinct sources: Family, Friends and a Significant Other—measured by three subscales of four items each. Items include “My family really tries to help me”, “I can count on my friends when things go wrong” and “There is a special person in my life who cares about my feelings”. Pregnant women were asked to rate the relevance of each statement to their feelings and experiences during the current pregnancy. Scores range from 12 to 84. Higher scores express stronger feelings of being socially supported [52]. This scale has also been validated among Lebanese adults [53]. In this study, Cronbach’s alpha = 0.956.

In addition to these measures, two scales were constructed, inspired by items retrieved from scales used in previous studies [54–56]. Factor analyses of these two scales are provided in Additional file 1. Both were scored based on a five-point response design, varying from “strongly disagree” (scored as 1) to “strongly agree” (scored as 5):

#### Social appearance concerns scale

In order to form a concise measure, four statements were selected from two distinct scales used in previous studies (the Pregnancy-Specific Questionnaire [54] and the Physical-Concern subscale of the Vanity scale [55]). The chosen items were “I am worried about how others view me (or will view me) during pregnancy”, “The way I look is extremely important to me”, “I would be ashamed or embarrassed if I were around people and did not look my best” and “Looking my best is worth the effort” [54, 55]. They incarnate deep preoccupations about maintaining a perfect corporal image in society. Higher scores indicate greater social appearance concerns. The Cronbach’s alpha was 0.788.

#### Media and pregnant celebrities influence scale

The aim of this scale was appraising to which extent the promotion of a thin and “ideal” pregnant body (via pregnant celebrities) on social media platforms could exert pressure on pregnant women. The following questions were included: “I like tracking what pregnant celebrities are doing”, “Seeing pregnant celebrities lose weight quickly after pregnancy is an inspiration to me”, “I feel pressured by the media and especially pregnant celebrities or celebrity moms to look thin during my pregnancy”, and “I like to copy what pregnant celebrities/stars wear in pregnancy” [54, 56]. They were selected from the Pregnancy-Specific Questionnaire [54] and the Celebrity Attraction Scale [56]. The impact of media and pregnant celebrities is greater on women with higher scores. The Cronbach’s alpha of those 4 items was 0.766.

### Statistical analysis

The data analysis and interpretation was performed using SPSS software version 25 (IBM, Armonk, NY, USA). To confirm the psychometric properties of the Social Appearance Concerns and Media and Pregnant Celebrities Influence scales, exploratory factor analyses were conducted on the items of both scales. Models’ adequacy was confirmed via the Kaiser–Meyer–Olkin (KMO) and Bartlett’s test of sphericity. Factors with Eigen values > 1 were retained.

The DEAPS score had a normal distribution since the skewness and kurtosis values varied between -1 and + 1 [57]. These conditions reinforce the assumptions of normality in samples larger than 300 [58]. Pearson correlation checked for linear correlation between continuous variables. The Student t-test and ANOVA F tests assessed categorical variables with two or more levels, respectively. Correlation coefficients between the absolute values of 0.1 and 0.23 are labelled as small correlations, between 0.24 and 0.36 as moderate, and greater than 0.37 as large [59]. Nagelkerke R^2^ revealed the explained variance of the dependent variable (A-DEAPS score) by the independent variables considered in the analysis model. All variables that showed a *p* < 0.25 in the bivariate analysis were taken as independent variables in the multivariable model [60], relying on the work of Bursac et al. and Pr. Hosmer and Pr. Lemeshow who state that these assumptions hold true for linear regressions. Since body dissatisfaction, social appearance concerns, and media/pregnant celebrities influence might seem to fall under a common category (i.e., appearance-related concerns), we wanted to ensure that no multicollinearity could exist in the regression model. The absence of multicollinearity was verified, with VIF values being < 2.5 [61].

### Mediation analysis

We used the PROCESS SPSS Macro version 3.4 model four [62]. to perform the mediation analysis, looking into a potential mediating effect of body dissatisfaction in the associations between disordered eating attitudes in pregnancy (i.e., the dependent variable) and each of the following independent variables: pregnancy-specific hassles, media and pregnant celebrities influence, and social appearance concerns. Independent variables showing a correlation coefficient or an effect size > │0.24│ in the bivariate analysis were incorporated in the mediation model, in order to get parsimonious models [59]. Mediation was only significant when the confidence interval (CI) around the indirect effect did not comprise zero [62]. Significance was considered achieved for a *p* < 0.05.

## Results

Our final sample was composed of 433 pregnant women, with a mean age of 28.55 ± 4.63 years and a mean pregnancy week (gestational age) of 23.68 ± 8.68. The A-DEAPS had a mean of 4.08 ± 2.78. Table 1 displays other characteristics and description of the total sample (Table 1).

**Table 1:** Sociodemographic characteristics of the participants

Variable	Total Sample (*N* = 433)
**Education level**	
Secondary or less	62 (14.3%)
University	371 (85.7%)
**Religion**	
Christian	107 (24.7%)
Muslim	326 (75.3%)
**Marital status**	
Married	433 (100%)
	**Mean ± SD**
**Age (in years)**	28.55 ± 4.63
**Household Crowding Index**	0.82 ± 0.44
**Physical Activity Index**	12.08 ± 14.48

### Bivariate analysis

The results of the bivariate analyses of factors associated with disordered eating are summarized in Tables 2 and 3. Higher current BMI, weight variation during pregnancy compared to before, depression, anxiety, pregnancy-specific hassles, body dissatisfaction, media and pregnant celebrities influence, and social appearance concerns were significantly associated with higher disordered eating attitudes in pregnancy (Tables 2 and 3). Moreover, a higher mean disordered eating attitudes score was found in women who are having their first pregnancy. On another hand, higher pregnancy-specific uplifts, perceived social support, and household crowding index were significantly associated with less disordered eating attitudes.

**Table 2:** Correlation between the disordered eating attitudes in pregnancy score and other continuous variables

Variable	Correlation Coefficient	*P*
Depression	0.263	**< 0.001**
Anxiety	0.233	**< 0.001**
Pregnancy-specific Uplifts	-0.221	**< 0.001**
Pregnancy-specific Hassles	0.445	**< 0.001**
Body Dissatisfaction	0.447	**< 0.001**
Perceived Social Support	-0.261	**< 0.001**
Media and Pregnant Celebrities Influence	0.361	**< 0.001**
Social Appearance Concerns	0.284	**< 0.001**

Numbers in bold indicate significant *p*-values

**Table 3:** Disordered eating attitudes in pregnancy score and sociodemographic characteristics

Variable	Mean ± SD	*P*	Effect size
**Education level**		0.539	0.074
Secondary or less	4.03 ± 2.76		
University	4.24 ± 2.89		
**Religion**		0.666	0.055
Christian	3.95 ± 2.63		
Muslim	4.10 ± 2.81		
**Gravidity – Number of the current pregnancy**		**0.009**	0.098
First pregnancy	4.36 ± 2.61		
Second pregnancy	3.76 ± 3.05		
Third pregnancy or more	3.97 ± 2.59		
	**Correlation Coefficient**	***P***	
Age	0.011	0.823	
Gestational Age	-0.09	0.058	
Household Crowding Index	-0.195	**< 0.001**	
Physical Activity Index	-0.048	0.324	
Current Body Mass Index	0.16	**0.001**	
Weight Variation during Pregnancy compared to before	0.16	**0.001**	

Numbers in bold indicate significant *p*-values

### Multivariable analysis

The results of a linear regression, taking the disordered eating attitudes in pregnancy score as the dependent variable, showed that higher pregnancy-specific hassles (B = 0.19), media and pregnant celebrities’ influence (B = 0.22), and body dissatisfaction (B = 0.17) were significantly associated with more disordered eating attitudes in pregnancy; whereas higher perceived social support (B = -0.03), higher household crowding index (i.e., lower socio-economic status) (B = -0.84), and multigravidity (i.e., having a third or more pregnancy compared to the first one) (B = -0.96) were significantly associated with less disordered eating attitudes during pregnancy (Table 4).

**Table 4:** Multivariable analysis: linear regression (ENTER method) taking the disordered eating attitudes in pregnancy score as the dependent variable

Variable	Unstandardized Beta	Standardized Beta	*P*	95% CI	VIF
Depression	-0.06	-0.11	0.090	-0.12; 0.01	3.29
Anxiety	-0.04	-0.11	0.120	-0.08; 0.01	3.82
Pregnancy-specific Uplifts	0.02	0.05	0.288	-0.02; 0.07	1.58
Pregnancy-specific Hassles	0.19	0.43	**< 0.001**	0.14; 0.25	2.63
Body dissatisfaction	0.17	0.30	**< 0.001**	0.12; 0.22	1.47
Perceived social support	-0.03	-0.18	**< 0.001**	-0.04; -0.01	1.46
Media and Pregnant Celebrities Influence	0.22	0.22	**< 0.001**	0.14; 0.30	1.27
Social Appearance Concerns	0.02	0.02	0.615	-0.05; 0.09	1.56
Gestational age	-0.01	-0.04	0.278	-0.04; 0.01	1.13
Household crowding index	-0.84	-0.17	**< 0.001**	-1.26; -0.42	1.32
Body mass index during pregnancy	0.01	0.03	0.467	-0.02; 0.04	1.23
Gravidity (second pregnancy vs first*)	-0.33	-0.06	0.162	-0.80; 0.13	1.25
Gravidity (third pregnancy or more vs first*)	-0.96	-0.14	**0.004**	-1.60; -0.31	1.57

^*^Reference group; numbers in bold indicate significant *p*-value; Nagelkerke R^2^ = 43.6%

### Mediation analysis

**Fig. 2:**
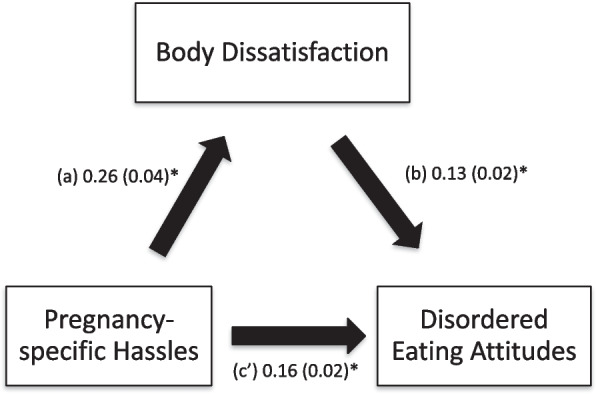
**a** Relation between pregnancy-specific hassles and body dissatisfaction; **b** Relation between body dissatisfaction and disordered eating attitudes; **c**’ Relation between pregnancy-specific hassles and disordered eating attitudes. Numbers are displayed as regression coefficients (standard error). *p < 0.001

**Fig. 3:**
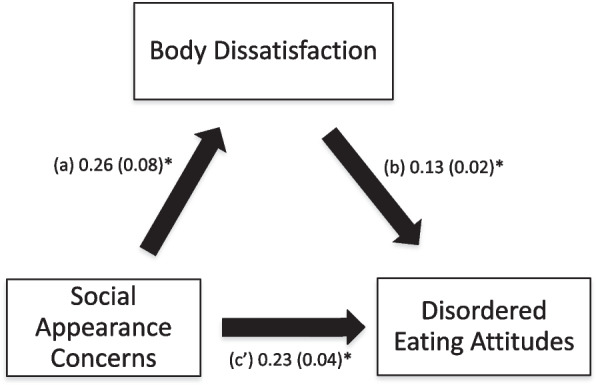
**a** Relation between social appearance concerns and body dissatisfaction; **b** Relation between body dissatisfaction and disordered eating attitudes; **c**’ Relation between social appearance concerns and disordered eating attitudes. Numbers are displayed as regression coefficients (standard error). *p < 0.001

The results of the mediation analysis are summarized in Table 5. Body dissatisfaction mediated the association between pregnancy-specific hassles and disordered eating attitudes (Fig. 2), and between social appearance concerns and disordered eating attitudes (Fig. 3).

**Table 5:** Mediation Analysis: Direct and indirect effects of the associations between independent variables, body dissatisfaction (mediator) and disordered eating attitudes in pregnancy

Independent Variable	Direct effect			Indirect effect		
	**Effect**	**SE**	***P***	**Effect**	**SE**	**95% BCa**
Pregnancy-specific Hassles	0.13	0.02	< 0.001	0.03	0.01	0.02–0.05
Media and Pregnant Celebrities Influence	0.05	0.04	0.295	-0.01	0.01	-0.04–0.01
Social Appearance Concerns	0.19	0.04	< 0.001	0.03	0.01	0.01–0.06

*Direct effect* Effect of the independent variable on disordered eating attitudes in the absence of the mediator (body dissatisfaction), *Indirect effect* Effect of the independent variable on disordered eating attitudes in the presence of the mediator (body dissatisfaction), *SE* Standard Error, *BCa* Bootstrap Confidence Interval

## Discussion

In this paper, we endeavored to provide a preliminary understanding of the “key paths” underpinning disordered eating attitudes during pregnancy. Our results demonstrated that the influence of the mass media, particularly through pregnant celebrities who have increasingly instantiated the “dream” pregnant body, was indeed a substantial contributive factor to disordered eating tendencies over the course of pregnancy. Body dissatisfaction turned out to be positively correlated with disordered eating as well. Certainly, the effects of social media, body image, and body dissatisfaction in potentiating eating disorders have always been spotlighted by the literature [28, 63, 64], with an increased vulnerability of the female audience. Recent Western studies have established significant associations between all these components and disordered eating attitudes among female college students and adolescents [27, 65].

More specifically, a study conducted among a population of celebrities-based tabloid magazine (CBTM) audience revealed that participants became excessively derogatory of the female pregnant body. In fact, those magazines have steadily conveyed standards of unrealistic fashionable pregnancies, creating convictions that women are supposed to remain thin at all times [66]. Consistently, a previous survey has shown that pregnant celebrities’ admiration stimulated excessive pregnancy weight-worry and body dissatisfaction among gravid women [56]. The issue belongs to the fact that when comparing themselves to celebrities, pregnant women experience the obsession of promptly restoring their pre-pregnancy states [67], hence engaging in weight-restrictive behaviors to outreach this purpose. Furthermore, it is acknowledged that body dissatisfaction during pregnancy may exasperate an existing or subliminal eating disorder [68]. Our findings also converge and add to the results of a recent study conducted among Lebanese pregnant women, reporting that high and addictive trends of social media posting during pregnancy intensified appearance comparisons and body dissatisfaction in this vulnerable population [69].

A further noticeable outcome was the significant positive association between pregnancy-related distress and disordered eating attitudes, in line with a previous study endorsing that pregnant women who reported increased weight-restrictive measures felt less excited and joyful towards their pregnancy [70]. The same study showed that self-consciousness and embarrassment towards weight gain during pregnancy were associated with magnified feelings of irritation and unpleasantness when confronting the usual stressors of pregnancy (e.g., thoughts about labor and baby, heartburn, etc.) [70]. In light of these facts, body dissatisfaction is likely to explain and potentiate the interaction between pregnancy-related hassles and disordered eating in pregnancy, through a synergistic mediation frame of action. Accordingly, the current research gave prominence to the mediating role of body dissatisfaction in the association between negative pregnancy-specific experiences and disordered eating attitudes during pregnancy.

On another hand, within a previous study conducted among college women, Menatti et al. established significant correlations between social anxiety and eating pathologies, including body dissatisfaction, drive for thinness, and bulimic symptoms. Moreover, fears of negative evaluation were identified as mediators of those relationships [71]. The latter study suggested that social interaction anxiety can be a major actor in the induction of body dissatisfaction and eating disorders, by instilling fears of being regarded by the society in a pejorative way [71]. These findings are apt to corroborate the mediating role that the present study has ascribed to body dissatisfaction within the relationship of social image concerns with disordered eating attitudes during pregnancy.

Further, our study supported the surmise that social support is a protective factor against disordered eating attitudes during pregnancy [5]. Consistently, in an interview-based study of pregnant women, testimonies revealed that partners' support, as well as positive comments from society regarding the pregnant body, were highly valued. Women raised the importance of discussions around body weight/image during antenatal visits [29], thus demonstrating that they all required information and support on these matters.

Furthermore, the present study discovered that multigravid women showed less propensities for disordered eating attitudes in pregnancy, contrasted to their primigravid peers. Our finding speculates that women having their first pregnancy would experience a high stress of dealing with pregnancy weight gain and body shape changes after delivery, thus apprehending their post-pregnancy physique and fearing of forever losing their pre-pregnancy body image. As a result, they are more vulnerable to adopt disordered eating attitudes, aiming at circumventing such body alterations. Indeed, the first pregnancy, in particular, is a time of great psychological turmoil, as women face a completely new situation, as well as a critical period in their growth as mothers, when significant changes in their physical appearance manifest [13].

Finally, in regard to socio-demographic characteristics, our analysis indicated that women pertaining to lower socio-economic households, reflected by a higher household crowding index [45], were less prone to disordered eating attitudes during pregnancy. Our result concurs with a previous Lebanese study conducted among the general population, which revealed that people with lower incomes tended to exhibit less trends of restrained eating habits [72]. This finding indicates that, especially amid the present Lebanese economic crisis, poor women would prioritize obtaining food, regardless of calories, over dieting and worrying about their weight gain during pregnancy.

### Clinical implications

Pregnancy is certainly a joyful period of life for the majority of women; however, some pregnant women may experience concealed issues with their maternal body image and pregnancy-related weight gain, hence indulging in risky eating patterns. Since stigmatization and poor professional abilities are the principal hindrances to depicting eating disorders during pregnancy and the post-natal period [73], the current study has addressed a significant gap in the detection of such pathology. Indeed, by elucidating the deep psychopathological patterns contributing to the phenomenon of disordered eating during pregnancy, our study provided significant knowledge into pathological weight-worry during pregnancy and its subsequent maladaptive behaviors such as calorie restriction, thus offering great advances to the psychopathological approach of women presenting with eating disorders during pregnancy. Moreover, the mediation analysis spotlighted body dissatisfaction as a potential mediator of the interactions between psychosocial factors, such as concerns about social appearance and pregnancy-related stress, and disordered eating among pregnant women. These findings may promote health care education in addition to guiding in the design of patient support programs. Namely, this study emphasizes the importance of confidential discussions about gestational weight gain, mental well-being, and body dissatisfaction in gynecology/obstetrics clinics. Targeted efforts should be deployed to identify women who are at risk for pregorexia, and treatment programs (e.g., psychological counseling, social support services, body acceptance programs, psychotherapy, or cognitive-behavioral therapy) must be enacted to assist them in achieving an adaptive perception of weight gain in the course of pregnancy as well as a healthy approach to their continuously changing pregnant bodies.

### Limitations and future research recommendations

Despite its contribution to the limited body of knowledge on disordered eating attitudes among pregnant women, our study has some limitations. For instance, its cross-sectional design strictly allows for a single point evaluation. As a result, it does not allow for causal and temporal inferences between variables of interest, nor does it ensure the assessment of these variables throughout the pregnancy trimesters. In this setting, our research focused solely on disordered eating attitudes during pregnancy. As such, the pre-pregnancy status of disordered eating was not assessed prior to the study's conduct, and it remains unclear whether the findings would have varied for women who had a history of dieting/diagnosed eating disorder prior to pregnancy compared to healthy peers. Further longitudinal research with control groups is warranted to address this issue and see if our findings can be replicated in other pregnant groups and if they fluctuate as women progress through the pregnant trimesters/months.

The symptoms of disordered eating were self-reported by the participants and not clinically diagnosed by a healthcare professional, making our results susceptible to a possible information bias. Although employing a pregnancy-specific instrument to appraise disordered eating symptoms, the hormonal/physiological changes in pregnancy cannot be controlled or quantified, resulting in a residual confounding bias. Mediation models in cross-sectional studies may also be problematic given that other confounders might interfere with the results. Furthermore, in the context of the COVID-19 pandemic, we employed the snowball technique to collect data on online networks, which might have predisposed us to a selection bias.

Finally, it is worth stating that the current study has scrutinized the drive for thinness and its associated weight-restrictive behaviors, which are referred to as pregorexia, within eating pathology during pregnancy. However, numerous facets of disordered eating in pregnancy remain understudied as well (e.g., binge eating, emotional eating, etc.), underscoring the utmost importance of further research to vigorously investigate other types of eating disorders among Lebanese pregnant women.

## Conclusion

In conclusion, our study highlighted that antenatal care, particularly in Lebanon, should no longer be limited to biological monitoring but rather seek to identify possible eating disorders and mental health threats. As a result of our findings, we prompt the implementation of national awareness campaigns and systematic antenatal screening programs for eating disorders in Lebanon, in order to bolster prenatal care and protect maternal mental health in our country. In this regard, thorough evaluation and monitoring of the factors associated with disordered eating in pregnancy, namely maternal distress, body dissatisfaction, and fascinations derived from the mediatization of gestational slimness, have become paramount. Further investigations following longitudinal designs should pursue identifying additional correlates of gestational disordered eating in the clinical context, in furtherance of consolidating screening programs and building targeted treatment strategies.

### Supplementary Information

**Additional file 1.** Factor analyses of the social appearance concerns and media and pregnant celebrities influence scales.

## Data Availability

All data generated or analyzed during this study are not publicly available due to restrictions from the ethics committee. The dataset supporting the conclusions is available upon request to the corresponding author.
