# Supplementary material for: Pregnancy through the Looking-Glass: correlates of disordered eating attitudes among a sample of Lebanese pregnant women
Source: BMC Psychiatry. 2023 Sep 26;23:699. doi: 10.1186/s12888-023-05205-w (PMC10521442; doi:10.1186/s12888-023-05205-w)
Supplement: Supplementary file 1 — Additional file 1. Factor analyses of the social appearance concerns and media and pregnant celebrities influence scales. [file 12888_2023_5205_MOESM1_ESM.docx]

**Factor Analyses of the Social Appearance Concerns and Media and Pregnant Celebrities Influence Scales**

All items in both scales were extracted and showed good psychometric properties.

| **Factor Analyses of the Social Appearance Concerns and Media and Pregnant Celebrities Influence Scales.** | | |
| --- | --- | --- |
| **Model 1:** **Social Appearance Concerns Scale** | | |
| **Item** | **Factor loading** | **H2 communalities** |
| 1- The way I look is extremely important to me | 0.840 | 0.642 |
| 2- I am worried about how others view me (or will view me) during pregnancy | 0.802 | 0.635 |
| 3- I would be ashamed or embarrassed if I were around people and did not look my best | 0.797 | 0.706 |
| 4- Looking my best is worth the effort | 0.717 | 0.515 |
| KMO=0.776; Bartlett test of sphericity p<0.001; explained variance=62.43%; Cronbach’s alpha=0.788. | | |
| **Model 2:** **Media and Pregnant Celebrities Influence Scale** | | |
| **Item** | **Factor loading** | **H2 communalities** |
| 1- I like tracking what pregnant celebrities are doing | 0.788 | 0.620 |
| 2- Seeing pregnant celebrities lose weight quickly after pregnancy is an inspiration to me | 0.757 | 0.572 |
| 3- I feel pressured by the media and especially pregnant celebrities or celebrity moms to look thin during my pregnancy | 0.748 | 0.559 |
| 4- I like to copy what pregnant celebrities/stars wear in pregnancy | 0.740 | 0.547 |
| KMO=0.681; Bartlett test of sphericity p<0.001; explained variance=57.47%; Cronbach’s alpha=0.766. | | |
